# Dysregulated TRAF3 and BCL2 Expression Promotes Multiple Classes of Mature Non-hodgkin B Cell Lymphoma in Mice

**DOI:** 10.3389/fimmu.2018.03114

**Published:** 2019-01-11

**Authors:** Gema Perez-Chacon, Magdalena Adrados, Maria T. Vallejo-Cremades, Sophie Lefebvre, John C. Reed, Juan M. Zapata

**Affiliations:** ^1^Instituto de Investigaciones Biomédicas “Alberto Sols”, CSIC-UAM, Madrid, Spain; ^2^Instituto de Investigación Hospital Universitario La Paz, Madrid, Spain; ^3^Instituto de Investigación del Hospital Universitario de La Princesa, Madrid, Spain; ^4^Sanford Burnham Prebys Medical Discovery Institute, La Jolla, CA, United States

**Keywords:** TRAF3, BCL2, DLBCL—diffuse large B cell lymphoma, plasma cell neoplasms, pathogen recognition receptors (PRRs), B cell lymphoma

## Abstract

TNF-Receptor Associated Factor (TRAF)-3 is a master regulator of B cell homeostasis and function. TRAF3 has been shown to bind and regulate various proteins involved in the control of innate and adaptive immune responses. Previous studies showed that TRAF3 overexpression renders B cells hyper-reactive to antigens and Toll-like receptor (TLR) agonists, while TRAF3 deficiency has been implicated in the development of a variety of B cell neoplasms. In this report, we show that transgenic mice overexpressing TRAF3 and BCL2 in B cells develop with high incidence severe lymphadenopathy, splenomegaly and lymphoid infiltrations into tissues and organs, which is the result of the growth of monoclonal and oligoclonal B cell neoplasms, as demonstrated by analysis of V_H_DJ_H_ gene rearrangement. FACS and immunohistochemical analyses show that different types of mature B cell neoplasms arise in *TRAF3/BCL2* double-transgenic (tg) mice, all of which are characterized by the loss of surface IgM and IgD expression. However, two types of lymphomas are predominant: (1) mature B cell neoplasms consistent with diffuse large B cell lymphoma and (2) plasma cell neoplasms. The Ig isotypes expressed by the expanded B-cell clones included IgA, IgG, and IgM, with most having undergone somatic hypermutation. In contrast, mouse littermates representing all the other genotypes (*TRAF3*-/*BCL2*-; *TRAF3*+/*BCL2*-, and *TRAF3*-/*BCL2*+) did not develop significant lymphadenopathy or clonal B cell expansions within the observation period of 20 months. Interestingly, a large representation of the HCDR3 sequences expressed in the *TRAF3*-tg and *TRAF3/BCL2*-double-tg B cells are highly similar to those recognizing pathogen-associated molecular patterns and damage-associated molecular patterns, strongly suggesting a role for TRAF3 in promoting B cell differentiation in response to these antigens. Finally, allotransplantation of either splenocytes or cell-containing ascites from lymphoma-bearing TRAF3/BCL2 mice into SCID/NOD immunodeficient mice showed efficient transfer of the parental expanded B-cell clones. Altogether, these results indicate that TRAF3, perhaps by promoting exacerbated B cell responses to certain antigens, and BCL2, presumably by supporting survival of these clones, cooperate to induce mature B cell neoplasms in transgenic mice.

## Introduction

Tumor Necrosis Factor Receptor (TNFR)-associated factors (TRAFs) constitute a family of scaffold proteins that interact with the cytoplasmic regions of various members of the TNFR superfamily upon their activation. TRAFs act as docking molecules for kinases, ubiquitin-ligases, ubiquitin-proteases and other effector proteins to comprise and modulate TNFR-signalosomes. In this regard, TRAFs regulate both the subcellular localization of the receptor-ligand complexes and the extent of the signaling response by controlling the composition and post-translational modification of proteins within these receptor signaling complexes. Additionally, some members of the TRAF family also regulate signaling and function of pattern recognition receptors (PRRs) and some interleukin-family receptors (1).

The role of TRAF-family proteins in regulating lymphocyte physiology and function is incompletely understood. This gap in knowledge is particularly relevant for TRAF3, which reportedly modulates multiple signaling pathways and plays a critical role in regulating B cell survival, activation and differentiation (2, 3). With regards to adaptive immunity, for example, TRAF3 binds and regulates signaling by different TNFR family members in B-lymphocytes, including CD40, B-cell activating factor receptor (BAFFR), transmembrane activator and CAML interactor (TACI) and B-cell maturation antigen (BCMA), which are critical regulators of B cell proliferation, differentiation and survival (4, 5). Additionally, TRAF3 regulates Toll-like receptors (TLRs) through its interaction with myeloid differentiation primary response 88 (MyD88) and TIR-domain-containing adapter-inducing interferon-β (TRIF) (6), thereby participating in innate immune responses against pathogen-associated molecular patterns (PAMPs) and damage-associated molecular patterns (DAMPs) (7, 8). TRAF3 is also involved in the regulation of nucleotide-binding oligomerization domain (NOD)-like receptors (NLRs) and retinoic acid-inducible gene (RIG)-1-like Receptors (RLRs) through its interaction with receptor interacting protein (RIP)-2 (9) and mitochondrial antiviral-signaling (MAVS) protein (10), respectively, which are intracellular sensors of bacteria and virus products (11). Moreover, TRAF3 has also been shown to regulate IL17R signaling (12). Thus, TRAF3 is a pleiotropic protein controlling multiple pathways involved in the regulation of B cell proliferation, differentiation, survival with broad relevance to both adaptive, and innate immunity.

Probably because of its pleiotropic effects, TRAF3 has seemingly opposite functions in some cellular contexts. This is well-illustrated by analysis of B cell-specific *Traf3*-deficient mice (13) and lymphocyte-specific *TRAF3*-tg mice (14). B cell-specific *Traf3*-deficient mice develop B cell hyperplasia and have high Ig titers in serum, suggesting that endogenous TRAF3 might suppress B-cell expansion. In this regard, *Traf3* deficiency in B cells results in nuclear factor κB (NF-κB)-2 activation due to a role of endogenous TRAF3 in recruiting ubiquitin ligases that promote degradation of NK-kB-inducing kinase (NIK) (15), although the actual mechanism involved in TRAF3-mediated NIK regulation in B cells remains controversial (16). One of the consequences of TRAF3 deficiency (presumably attributed to the NF-kB2 over-activation) is the expansion of marginal zone (MZ) B cells (13, 17), which might explain the hyperreactivity to TLR ligands (18) and the systemic lupus erythematosus (SLE)-like autoimmunity observed in these mice (13). MZ B cells do not normally express or have very reduced levels of TRAF3 expression (19) and are naturally overreactive to TLR ligands (11, 20). In contrast, lymphocyte-specific *TRAF3*-tg mice develop progressive plasmacytosis and hypergammaglobulinemia, show exacerbated TLR responses as well as increased IgG production in response to T-I and T-D antigens, and develop systemic inflammation and SLE-like autoimmunity (14). This phenotype suggests that TRAF3 over-expression also causes excessive B-cell function that can manifest as SLE-like autoimmunity, in this case perhaps by driving B cell differentiation to produce abundant antibody-secreting cells (ASCs) via a process that might speculatively be PRR-dependent.

TRAF3 has been proposed as a tumor suppressor protein since a number of biallelic deletions or inactivating mutations has been identified in human B cell malignancies, including B-chronic lymphocytic leukemia (CLL), splenic marginal zone lymphoma (SMZL), mantle cell lymphoma, diffuse large B-cell lymphoma (DLBCL), and multiple myeloma (MM), as well as in Waldenström's macroglobulinemia (21–27). In agreement with these results, B-cell-specific *Traf3*-deficient mice were reported to develop clonal SMZL or B1a lymphomas (28). These results are consistent with the hypothesis that TRAF3 inactivating mutations (resulting in constitutive NF-κB2 activation in B cells) predispose to malignant transformation irrespective of the B cell maturation state.

Previously, we have shown that lymphocyte-specific *TRAF3-*tg mice have extra-nodal infiltration of B-cells into many organs and these animals experience an increased incidence of inflammation-driven solid tumors, including squamous cell carcinomas, lung carcinomas and hepatomas. However, these mice did not show formal evidence of B-lymphoid malignancy. Interestingly, intraperitoneal (I.P.) inoculation of pristane, a natural saturated terpenoid alkane known to promote autoimmune diseases and plasmacytoma in mice (29, 30), into *TRAF3*-tg mice resulted in increased tertiary organs formation and exacerbated autoimmunity, but other than a few cases of plasmacytoma, this treatment failed to promote manifest development of myeloma or other B-cell neoplasms (14). Taken together, these results suggest that TRAF3 upregulation causes severe alterations in B cell differentiation but is not sufficient to promote B cell transformation by itself.

Previously, we reported that mice with the combination of upregulated BCL2 and deficient TRAF2 signaling in B cells develop small B cell lymphoma (SBL)/CLL with high incidence, while neither deficient TRAF2 function nor BCL2 upregulation alone were sufficient to induce CLL or other malignancies in mice (31, 32). BCL2 overexpression is a landmark of CLL, follicular lymphoma (FL) and other B cell malignancies (33), including DLBCL (34). In this report, we show that the combination of TRAF3 and BCL2 overexpression in B cells leads over time to severe lymphadenopathy, splenomegaly and extranodal lymphoid infiltrations in tissues and organs in the mice, which is not observed in mice with mono-transgenic TRAF3 or BCL2. This dysplasia is the result of monoclonal and oligoclonal B cell neoplasms (as demonstrated by the analysis of rearranged *V*_*H*_*DJ*_*H*_ genes). In addition, we show that TRAF3 upregulation favors the production of *V*_*H*_*DJ*_*H*_ rearrangements producing HCDR3 sequences similar to those recognizing PAMPs and DAMPs.

## Materials and Methods

### Transgenic Mice

Lymphocyte-specific *TRAF3*-tg (14) and B cell-specific *BCL2*-tg mice mimicking the t(14;18) (q32;21) chromosomal translocation involving *BCL2* and *IgH* found in human FLs (35) have been previously described. *TRAF3*-tg (FVB/N) and *BCL2*-tg (BALB/c) heterozygous mice were bred to produce F1 litters with progeny of the four possible genotypes [(wild-type –/–; *TRAF3*-tg (single-positive +/–); *BCL2*-tg (single-positive –/+); and *TRAF3/BCL2* (double-positive +/+)] expressed on FVB/N x BALB/c mixed background. Analysis of the transgenic mouse genotypes was performed by polymerase chain reaction (PCR) using primers specific for human TRAF3 (forward 5′-TCGAGTTTGCCACCATGG-3′ and reverse 5′-GCGCGATCATCGGAACC-3′) and BCL2 (forward 5′-TTAGAGAGTTGCTTTACGTGGCCTG-3′ and reverse 5′-ACCTGAGGAGACGGTGACC-3′). The animal protocols were approved by the Institutional Animal Care and Use Committees of the Sanford Burnham Prebys Medical Discovery Institute and by the Bioethics Committee of the Consejo Superior de Investigaciones Científicas. Mice showing symptoms of distress and pain (heavy breath, weight loss, lethargy, etc.) were euthanized. All transgenic mice in the study were heterozygotes for each transgene.

### Antibodies

Antibodies against human TRAF3 (19) and BCL2 (36) were previously described. TRAF3 (C-20), CD10 (F-4), BCL6 (N-3), PCNA (FL-261), and ERK2 (C-14) were from Santa Cruz Biotechnologies. MUM-1 (ABIN721195, antibodies online), CD45R/B220 (14-0452-81, Thermofisher scientific), Ki67 (Ab15580, Abcam), cIAP1/2 (R&D systems) and pre-adsorbed HRP-conjugated anti-mouse IgG (Sigma-Aldrich) and anti-mouse IgA (Novus biologicals) were used for western blot and/or immunohistochemistry analysis. Anti-rabbit and anti-mouse HRP-conjugated secondary antibodies were from Santa Cruz Biotechnologies or from Sigma-Aldrich. For flow cytometry analysis FITC- PE- and APC-labeled antibodies against mouse CD45R/B220, CD19, CD21, CD23, CD5, CD43, CD138/Syndecan-1, IgM, IgD, IgG (all from BD Biosciences) were used.

### Isolation of Mononuclear and B Cells

Spleens, lymph nodes and blood from tg mice and WT littermates were collected and mononuclear cells were isolated by Ficoll density centrifugation (Lympholyte-M; Cedarlane Laboratories, Burlington, NC). B cells were isolated by negative magnetic selection using the StemSep mouse B cells enrichment kit (StemCells Technologies, Vancouver, CA), following the manufacturer's specifications.

### Flow Cytometry Analysis

Mononuclear cells isolated as described earlier were incubated with 50 μg/ml human γ-globulin for 10 min at 4°C. Then, 10^6^ cells were incubated with a combination of FITC-, PE-, or APC-conjugated antibodies recognizing various surface markers. After 40 min of incubation at 4°C, cells were washed with PBS and analyzed by flow cytometry in a FACS Canto II cytofluorimeter and the FlowJo (LLC) and FACSDiVa 6.1.2 (BD Biosciences) cytometry analysis softwares. Intracellular IgG expression was determined using a commercial fixation/permeabilization kit (Fitx&Perm; Invitrogen Life Technologies), following the manufacturer's instructions.

### Immunohistochemistry

Tissues and organs from transgenic mice were fixed in 10% formalin (Sigma-Aldrich), embedded in paraffin. Tissue sections (5 μm) were deparaffinized and antigen retrieval was then performed in citrate buffer solution pH 6 (Dako). Sections were then rinsed with distilled water, treated 10 min at room temperature with peroxidase blocking solution (10% H_2_O_2_ in methanol) and then washed with TBS. After blocking with a TBS buffer containing normal goat serum for 1 h at room temperature, the corresponding primary antibodies were applied to the sections over night at 4°C. After washing with TBS, a HRP conjugated secondary antibody (Sigma Aldrich) was used to detect the primary antibody. Color was developed using a diaminobenzidine-based detection method (Vector Laboratories, Burlingame, CA), and sections were then counterstained with hematoxylin, dehydrated, and mounted in DPX (Fluka). Tissue sections were stained with hematoxylin and eosin (H&E).

### Immunoblots

Cells from different mouse tissues were lysed in modified Laemmli buffer (0.125 M Tris pH 6.8, 4% SDS, and 20% glycerol) and sonicated. Protein concentration was determined by the bicinchoninic acid method (Pierce, Rockford, IL). Protein samples (8–15 μg/sample) were supplemented with 2.5% 2-mercaptoethanol and 0.004% bromophenol blue, and subjected to SDS-PAGE analysis and immunoblotting, using the indicated primary antibodies and horseradish peroxidase-conjugated secondary antibodies. Specific bands were detected using enhanced chemiluminescence and exposure on film. ERK2 expression was used as an internal loading control.

### *V_*H*_DJ_*H*_* Analysis

Tissues and cells from *TRAF3xBCL2* mice representing all different genotypic combinations (–/–; +/–; –/+; and +/+) were extracted and total RNA was isolated using TRI_ZOL_ reagent and the PureLink^TM^ RNA mini kit (Life Technologies, Carlsbad, CA), following the manufacturer's instructions. Then, RNA was reverse transcribed into cDNA using 2 U Superscript II reverse transcriptase (Life Technologies). The *IGHV* regions were amplified using the following primers, VH primer (forward) 5′-SARGTBMAGCTGSAGSAGTCWGG-3′; CHμ primer (reverse) 5′-CAGATCTCTGTTTTTGCCTCGTA-3′; CHγ primer (reverse) 5′-ATGCAAGGCTTACACCACAATCC-3′; and CHα primer (reverse) 5′-TAATAGGAGGAGGAGGAGTAGGAC-3′ (Life Technologies). After denaturing DNA at 94°C for 10 min, the PCR conditions entailed 38 cycles of denaturing at 94°C for 1 min, annealing at 52°C for 1 min and extension at 68°C for 1 min, with a final extension step at 68°C for 10 min. The PCR products were then analyzed by gel electrophoresis in a 2% agarose gel, excised and purified (Qiagen). Purified products were cloned using the pGEM®-T Vector System (Promega, Madison, WI, USA) and transformed into bacteria, following the manufacturer's instructions. From 5 to 15 bacterial colonies of each sample were grown in culture overnight and the plasmids were extracted using the Wizard® Plus SV Minipreps DNA Purification System (Promega). Sequencing was performed by GATC Biotech (Konstanz, Germany). Nucleotide sequences were analyzed by means of Chromas 2.4.3 software (Technelysium, Queensland, Australia) and compared with those mouse germ-line sequences available in the IMGT/V-QUEST databases (37, 38). Sequences with < 97.5% identity to the corresponding germ-line *IGHV* sequence were considered as mutated. Isolectric point (pI) of HCDR3 region was calculated with the Compute pI/Mw tool (ExPASy Bioinformatics Resource Portal, http://web.expasy.org/compute_pi/). HCDR3 analysis was carried out comparing the sequence in the protein BLAST database (restricted to *Mus musculus* sequences). Only sequences with at least 75% identity were considered.

### Adoptive Transfer

Splenocytes or lymphocytes isolated from lymph nodes, ascites or pleural effusion (40–60 × 10^6^) from representative *TRAF3/BCL2* double-tg mice with lymphoma were I.P. allo-transplanted into 8–12 weeks-old non-obese diabetic/severe combined immunodeficiency (NOD/SCID) mice. Animals were euthanized when the mice develop sign of illness (distended belly, respiratory distress, lethargy, etc).

### Statistical Analysis

Survival analyses were performed using the Kaplan-Meier method and the log-rank test. Differences were regarded as significant when *p* < 0.05.

## Results

### *TRAF3/BCL2* Double-tg Mice Develop Severe Lymphoid Dysplasia and Have a Reduced Lifespan

**Figure 1:**
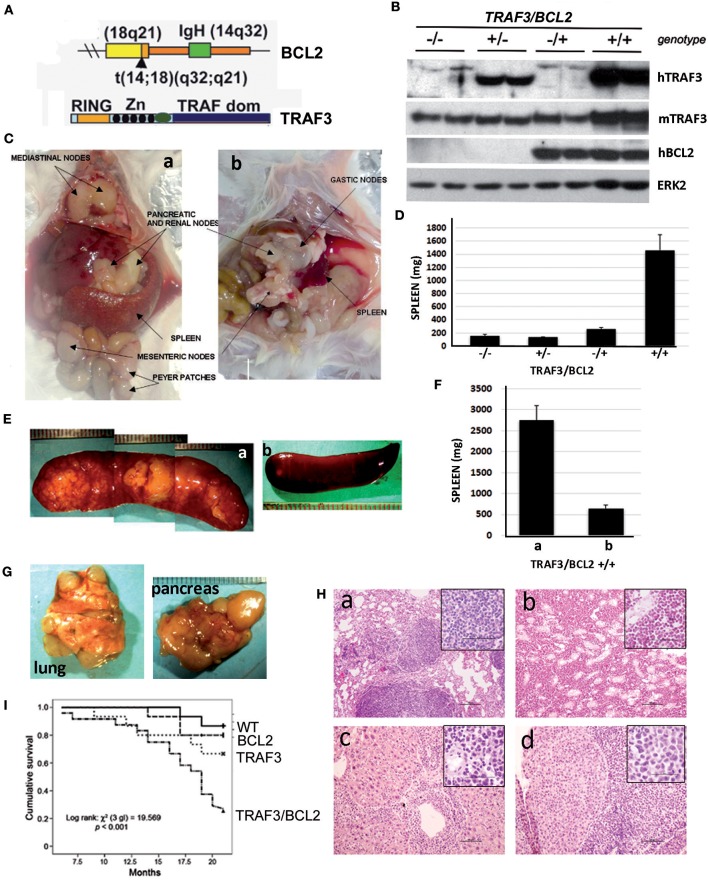
*TRAF3/BCL2* double-tg mice develop severe lymphoid dysplasias and have a reduced lifespan. **(A)** Schematic representation of the expressed transgenes. *BCL2* gene mimics the t(14;18)(q32;q21) translocation involving BCL2 and IgH found in human FLs resulting in BCL2 overexpression. *TRAF3* is under the control of the IgH promoter and the μ enhancer. **(B)** Representative examples of the TRAF3 and BCL2 expression in spleen extracts from mice with different *TRAF3/BCL2* genotypes. Expression of ERK2 is used as loading control. **(C)** Representative examples of *TRAF3/BCL2* double-tg mice with lymphoid dyscrasias. Mice usually develop two types of lymphadenopathies, group 1 characterized by massive splenomegaly and disseminated lymphadenopathy **(a)** and group 2 with moderate splenomegaly and disseminated lymphadenopathy **(b)**. **(D)** Weight of the spleens of mouse littermates with the different *TRAF3/BCL2* genotypes (–/–, *n* = 7; +/–, *n* = 10; –/+, *n* = 10; +/+, *n* = 32). Mice were euthanized when +/+ mice developed lymphoid dyscrasias. Data represent mean ± SEM. **(E)** Representative examples of the enlarged spleens developed by diseased *TRAF3/BCL2*-double-tg mice. The pictures illustrate the differences in aspect and morphology of group 1 **(a)** and group 2 **(b)** spleens. **(F)** Weight of the spleens of group 1 [**(a)**, *n* = 13] and group 2 [**(b)**, *n* = 19]. Mice were euthanized when +/+ mice developed lymphoid dyscrasias. Data represent mean ± SEM. **(G)** Representative examples of lungs (left) and pancreas (right) showing prominent lymphoid metastasis from *TRAF3/BCL2*-double-tg mice. **(H)** H & E staining of tissues from representative *TRAF3/BCL2*-double-tg mice showing lymphoid infiltrations. Figure shows lungs **(a)**, kidney **(b)**, liver **(c)**, and pancreas **(d)**. Magnification is 100 x. The square inside shows a 400 x magnification capture of the infiltrating lymphocytes. Scale bars are shown. **(I)** Kaplan–Meier analysis of survival of mice with the different *TRAF3/BCL2* genotypes (–/–, *n* = 15; +/–, *n* = 15; –/+, *n* = 15; +/+, *n* = 24). Survival analysis was performed by using the nonparametric model of Kaplan–Meier. Log-rank test analysis of *TRAF3/BCL2* double-tg mice survival compared to the other groups demonstrated statistical significance (vs. wild-type, 0.0005; vs. TRAF3-tg, 0.029; vs. BCL2-tg, 0.002).

To assess whether TRAF3 upregulation might contribute to B cell transformation, we crossed lymphocyte-specific *TRAF3*-tg mice (14) with B cell-specific *BCL2*-tg mice (35). A schematic representation of the expressed transgenes is shown in Figure 1A. *TRAF3*-tg (FVB/N background) and *BCL2*-tg (BALB/c background) mice were crossed to produce F1 litters with mice harboring the different transgene combinations, *TRAF3/BCL2* –/–, +/–, –/+, and +/+. Immunoblot analysis of spleen extracts from mice bearing the *TRAF3* and *BCL2* transgenes readily demonstrated the expression of TRAF3 and BCL2. Moreover, hTRAF3 did not alter the expression of endogenous mTRAF3 (Figure 1B). Young *TRAF3/BCL2* double-tg mice did not show any significant alteration other than modest splenomegaly, which was similar to that of the *BCL2*-tg mice (31). However, when *TRAF3/BCL2* double-tg mice became older they began developing severe lymphoid dyscrasias, characterized by massive splenomegaly, and overt disseminated lymphadenopathy (Figure 1C). Some of the mice also develop pleural effusions and ascites. In contrast, these pathologies were not found in littermates of the other genotypes as they aged (Figure 1D and data not shown).

Interestingly, we observed a pattern in the size and shape of the spleens of the *TRAF3/BCL2* double-tg mice that developed lymphoid pathologies. Many spleens were extremely large, ranging from 1.5 to 5.8 g, with a pale appearance suggestive of an accumulation of white blood cells disproportionally to red blood cells. The spleens grossly had a lumpy surface and patchy decolorized zones suggestive of large lymphoid nodules (Figures 1E,Fa). Alternatively, spleens from some animals were larger than normal (0.3–1.2 g) but with a grossly normal appearance (Figures 1E,Fb).

Lymphadenopathy could be found in the double transgenic mice irrespective of gross splenic morphology (Figure 1C). In addition, diseased *TRAF3/BCL2* double-tg mice show massive lymphoid infiltrations in a variety of organs that often could be seen on gross pathological examination (Figure 1G). Histopathology studies confirmed the severe lymphoid infiltration of various tissues and organs (Figure 1H), including lung (Figure 1Ha), kidney (Figure 1Hb), liver (Figure 1Hc), and pancreas (Figure 1Hd). Consistent with the lymphoproliferative pathology observed in the *TRAF3/BCL2* double-tg mice, these animals also have a significantly shorter lifespan than their littermates with wild-type, *BCL2*-tg, and *TRAF3*-tg genotypes (Figure 1I).

### DLBCL and Plasma Cell Neoplasms Are the Most Common Types of B Cell Dyscrasias Developed by *TRAF3/BCL2* Double-tg Mice

**Figure 2:**
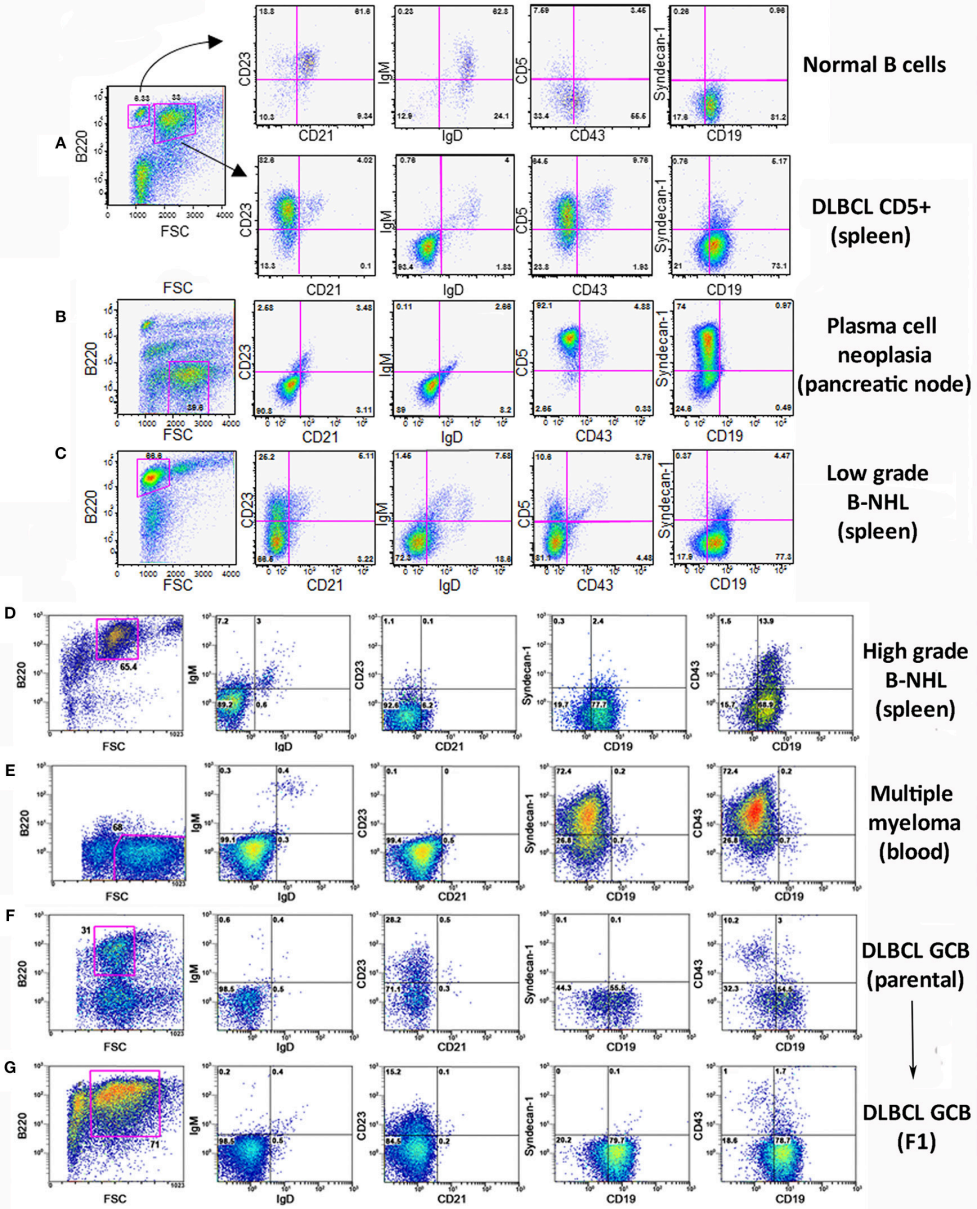
Analysis of the B lymphocyte populations expanded in *TRAF3/BCL2* double-tg mice with lymphoid dyscrasias. Three-color flow-cytometry analysis was performed to determine the phenotype of expanded B lymphocyte populations. Gating of the expanded population was based on the CD45R/B220 and FSC plot of each sample analyzed and is indicated in the figure. The surface molecules analyzed are indicated in the plots, as well as the percentage of cells found in each quadrant. The quadrants settings were selected based on the staining of isotype-controls (not shown). The tissue source where the analyzed lymphocytes were extracted from and the type of B cell malignancy developed by the *TRAF3/BCL2* double-tg mice, according to the flow-cytometry and immunohistochemical analysis, is indicated in the figure.

Flow cytometry analysis of the lymphoid populations from lymphoid tissues of diseased *TRAF3/BCL2* double-tg mice, including spleen, nodes, blood, as well as ascites, and pleural effusion when found in the mice, showed that they were consistently composed by B cell expansions with distinct surface marker expression but all indicative of a mature B cell phenotype. Representative examples are shown in Figure 2. Our results indicated that the vast majority of these B cell populations could be allocated into two major groups. The first group was characterized by large cells (FSC^H^) expressing CD45R/B220 and CD19, but having lost surface IgM, IgD, and CD21 expression (Figures 2A,D,F,G). The other group was composed by large cells lacking CD45R/B220, CD19, CD21, CD23, IgM, and IgD on their surface but expressing syndecan-1 (CD138) (Figures 2B,E, 4A), which is indicative of plasma cell lineage. In addition, a few mice developed a type of lymphoid expansion composed by small B cells expressing CD45R/B220 and CD19 and lacking the expression of CD21, CD23, IgM, and IgD on their surface (Figure 2C). For comparison, Figure 2A, top, shows the surface markers expression analysis of the remaining normal B cell population present in the spleen of that mouse.

**Figure 3:**
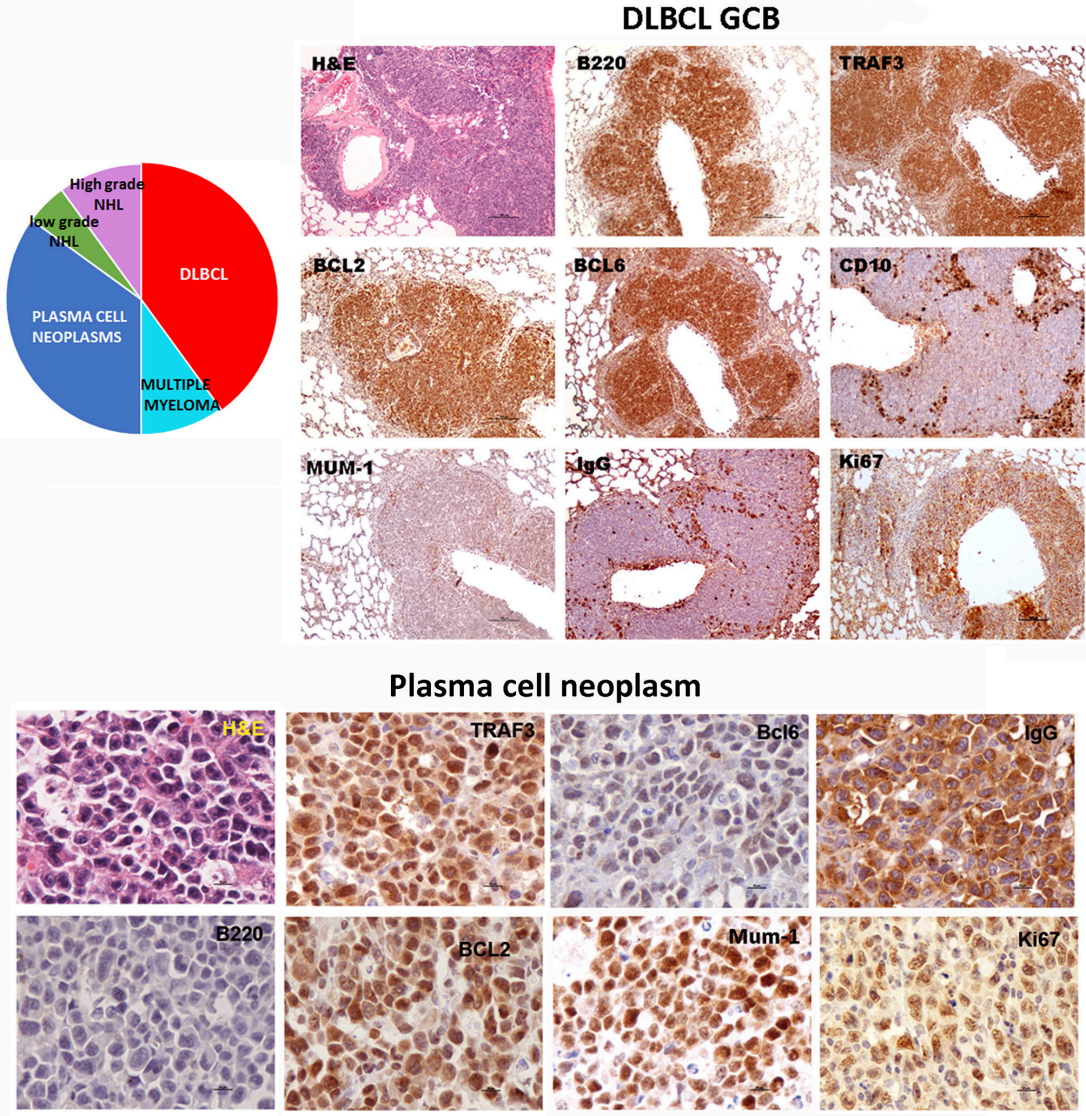
*TRAF3/BCL2* double-tg mice develop different types of mature B cell neoplasias, but predominantly DLBCL and plasma cell neoplasias. Immunohistochemical analysis of representative examples of a DLBCL GCB type and a plasma cell neoplasia developed by the *TRAF3/BCL2* double-tg mice. Tissue slides were stained either with H&E or with antibodies specific for human TRAF3 and BCL2 and for mouse CD45B220, BCL6, MUM-1, CD10, IgG and Ki67, as indicated. Scale bars are 100 μm (DLBCL) and 10 μm (plasma cell neoplasia). A diagram indicating the frequency of the different lymphoid neoplasias found in the *TRAF3/BCL2* double-tg mice is shown (*n* = 18).

**Figure 4:**
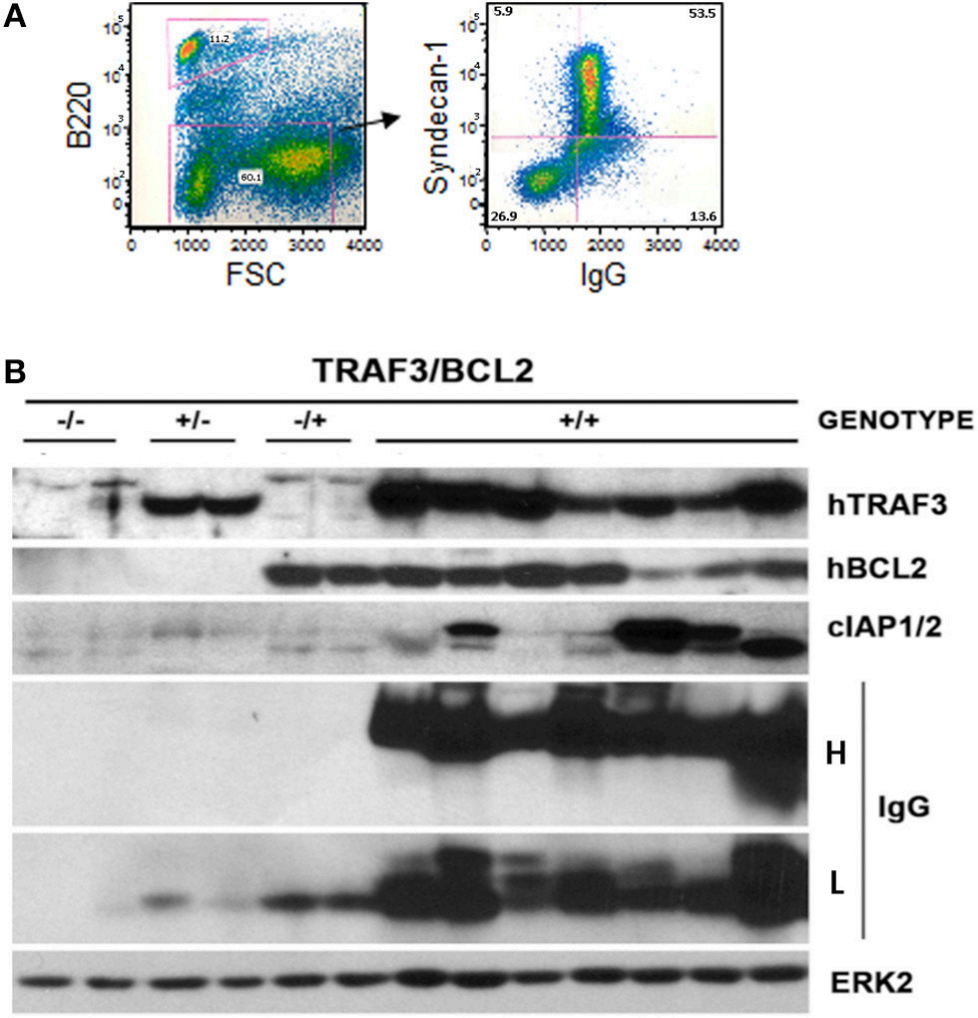
Development of plasmablastic B cell neoplasms by *TRAF3/BCL2* double-tg mice. **(A)** Flow cytometry analysis of a representative *TRAF3/BCL2* double-tg mouse showing the expansion of an FSC^high^ CD45B220^null^ lymphoid population with surface syndecan-1 (CD138) and cytosolic IgG expression. **(B)** Western blot analysis of lymphoid extracts (spleens and nodes) of representative examples of TRAF3/BCL2 double-tg (+/+) mice that have developed plasma cell neoplasms. For comparison, 2 representative examples of spleen extract from wild-type (–/–), T*RAF3*-tg (+/–), and *BCL2*-tg (–/+) mice is also shown. The expression of human TRAF3 and BCL2, and mouse cIAP1/2 and IgG heavy H and light L chains is shown. ERK2 expression is shown as loading control.

Sequencing of the *V*_*H*_*DJ*_*H*_ region of the heavy chain (*IgH*) gene locus (deduced from the transcriptome) showed that both the large B-cell and the plasma cell types of lymphoid expansions were either monoclonal or oligoclonal (Table 1), thus indicating that the *TRAF3/BCL2* double-tg mice develop lymphoid neoplasms. Further characterization of the B cell neoplasms developed by these mice was accomplished by immunohistochemistry. These results further confirm the expression in these neoplasms of TRAF3 (which was more often located in the nucleus than in the cytoplasm), and BCL2 (which was present in the cytosol) (Figure 3 and Supplementary Figures 1–3). Moreover, based on the differential expression of MUM-1, BCL6, and CD10, we conclude that most of the lymphoid neoplasms characterized by very large spleens were consistent with DLBCL. Figure 3 shows a DLBCL expressing BCL6^+^, MUM-1^null^ and CD10^null^, representative of the GC B cell cluster. Other examples of DLBCL are shown in Supplementary Figure 1. In the DLBCL group, which is characterized by large cells (FSC^H^) expressing CD45R/B220 and CD19, we also found examples of mice with B cell neoplasms showing a prominent starry sky pattern, positive staining for BCL6, MUM-1, and CD10 and a high proliferation index, as shown by Ki67 staining, consistent with a high-grade B-non Hodgkin lymphoma (NHL) (Supplementary Figure 2). Of note is that this later lymphoma also has c-MYC overexpression (not shown). Interestingly, one mouse developed a B cell neoplasm consistent with a rare type of DLBCL expressing CD5 (Figure 2A). Immunohistochemical analysis of representative examples of the plasmacytoid neoplasms confirmed expression of either cytosolic IgG or IgA and showed a high Ki67 proliferation index (>50%), consistent with a plasma cell neoplasia (Figure 3 and Supplementary Figure 3). Consistent with this diagnosis, some neoplasms of this group also express cytosolic IgG as demonstrated by FACS and immunoblotting of protein extracts of lymphoid tissues from representative *TRAF3/BCL2* double-tg mice with this type of lymphoid expansions (Figure 4). A diagram representing the frequency of the different B cell neoplasms found in the *TRAF3/BCL2* double-tg mice is provided in Figure 3.

**Table 1:** TRAF3/BCL2 double-tg mice develop clonal B cell expansions.

**Mouse ID**	**Tissue**	**Ig class**	**IGHV family**	**IGHD family**	**IGHJ gene**	**% Homology**	**SHMs**	**Clone (%)**	**Funcionality**	**HCDR3**	**pI**	**Tumor type**
TRAF3/BCL2 3 Parental	Ascitis	IgA	VH3	D2	JH2	93.8	M	62	Productive	ASRYGLFDY	5.88	HIGH GRADE B-NHL
	Node	IgM	VH12	D2	JH1	97.6	NM	40	Productive	AGDSDGYWYFDV	3.42	Not determined
TRAF3/BCL2 11	Node+	IgM	VH1	D2	JH4	94.4	M	100	Productive	AREPYGDYDAMDY	3.84	Plasma cell neoplasia
TRAF3/BCL2 22	Node	IgA	VH1	D2	JH3	91.3	M	100	Productive	AREDYYAWFAY	4.37	Plasma cell neoplasia
TRAF3/BCL2 25	Spleen	IgG	VH1	D2	JH4	86.1	M	80	Productive	ARSRSGVTYFYYTLDF	8.54	DLBCL
TRAF3/BCL2 39	Node	IgA	VH2	D2	JH4	96.14	M	100	Productive	AKHRYDAMDY	6.79	Plasma cell neoplasia
TRAF3/BCL2 53a	Spleen	IgG	VH1	D6	JH4	91.7	M	78	Productive	TRQSHYAMDY	6.41	DLBCL
	Node	IgG	VH1	D2	JH4	95.8	M	100	Productive	ARRGYDGAMDY	6	Not determined
TRAF3/BCL2 73	Blood	IgM	VH2	D3	JH3	97.5	NM	100	Productive	ASQGY	5.57	Multiple myeloma
TRAF3/BCL2 79	Node	IgG	VH3	D1	JH4	97.22	M	100	Productive	ALRGDY	5.88	plasma cell neoplasia
TRAF3/BCL2 20	Spleen	IgM	VH5	D4	JH1	96.9	M	100	Productive	ARLDWYFDV	4.21	DLBCL
	Pleural effusion	IgM	VH5	D4	JH1	96.2	M	100	Productive	ARLDWYFDV	4.21	
TRAF3/BCL2 26	Node	IgG	VH14	D2	JH2	90.97	M	80	Productive	ASDYDCLGF	3.56	Not determined
TRAF3/BCL2 32	Node	IgG	VH1	D1	JH3	98.26	NM	80	Productive	TRSDFYGPWFAY	5.5	DLBCL
TRAF3/BCL2 36	Node	IgA	VH5	D1	JH4	98.61	NM	100	Productive	ARHKKAMDY	9.7	DLBCL CD5+
TRAF3/BCL2 40	Node	IgG	VH1	D1	JH1	88.2	M	40	Productive	GRVGHYYGFHLFFDV	6.92	DLBCL
TRAF3/BCL2 53b	Spleen	IgM	VH3	D2	JH2	95.8	M	43	Productive	AREKDTDGYYFDY	4.23	DLBCL

*Representative examples of the expanded B cell clones found in TRAF3/BCL2 double-tg mice. In the table is indicated the ID number of the mouse, the source of the mRNA sample, the Ig class of the expanded clone is also indicated and highlighted (IgM, green; IgG, blue; IgA, red). The IGHV, IGHD, and IGHJ families that were reorganized in the clone are indicated, according to IMGT/V-QUEST database. The percentage of sequence homology of the VDJ sequence of each clone compared to that of the germinal sequence (GC) is also indicated (% homology) and whether the clone meets the criteria of having been subjected to somatic hypermutation (SHMs) (non-mutated (NM) ≥ 97.5 identities with the GC sequence; mutated (M) < 97.5% identities with the GC sequence). The percentage of the VDJ sequences analyzed from a given sample corresponding to the expanded clone is indicated (% clone). Functionality refers to whether the expanded clone encoded a productive Ig or contained internal stop codons (unproductive). The HCDR3 sequence is also provided. Basic (red) and acid (green) amino acids are highlighted and the isoelectric point (pI) of the HCDR3 sequence is shown. The B cell neoplasia developed by these representative TRAF3/BCL2 double-tg mice is indicated. The diagnostic was based on macroscopic and microscopic features, flow cytometry and immunohistochemical analysis. Mouse 53a and 53b come from two different and independent colonies and have different parents*.

### *TRAF3/BCL2* Double-tg Mice Develop Clonal B Cell Expansions

As indicated above, the analysis of the *V*_*H*_*DJ*_*H*_ rearrangements confirmed the existence of clonal B cell expansions in the *TRAF3/BCL2* double-tg mice observed with aging (Table 1). The expanded clones were mono- or oligoclonal and the Ig subtypes of these clones varied (IgA, IgG, and IgM were observed). In addition, somatic hypermutation (SHM) took place in approximately half of the IgM clones and most (75–80%) of the IgA and IgG clones. The fact that most of these clones have experienced Ig class switching and SHM suggests that these neoplasms arise from antigen-activated B cells that have undergone differentiation in germinal centers (GCs), although extra-follicular differentiation is also a possibility, in particular for those clones expressing IgM. In some instances, distinct clonal expansions were found in different lymphoid tissues of the same mouse, as indicated by the Ig subclass and the HCDR3 sequence of the expanded clones (Table 1).

As one hallmark of cancer is the ability of tumor cells to grow into immunodeficient recipients after transplantation, we used splenocytes or lymphocytes from either ascitic fluid or pleural effusion for allo-transplantation into SCID/NOD immunodeficient mice (Table 2). For these experiments we used lymphocytes from mice representing three of the most characteristic of the B cell neoplasms developed by the *TRAF3/BCL2* double-tg mice. One of the donor mice (#3) developed splenomegaly (1,200 mg), severe diffuse lymphadenopathy (5,000 mg) and ascitic fluid. FACS analysis (Figure 2D) and immunohistochemistry (Supplementary Figure 2) of the spleen was consistent with a high-grade B-NHL. A major IgA clone was found in spleen and ascites, although other polyclonal IgM, IgG, and IgA populations were also found, in particular in ascitic fluid (Supplementary Figure 4). In addition, the analysis of a node from this mouse showed the existence of an expanded clone (IgM) different to that found in spleen and ascites (Table 1). Allo-transplantation of ascitic lymphocytes from mouse #3 resulted in efficient implantation, taking only 3–4 weeks for the development of overt lymphoma in recipient mice. Necropsies showed that recipient SCID/NOD mice had massive lymphadenopathy and ascitic fluid but fairly normal spleens (Table 2). A similar result was obtained when ascites from one F1 transplanted mouse was transferred to another SCID/NOD recipient (F2). The analysis of the IgM, IgG, and IgA populations in the recipient mice showed a striking enrichment of the IgA population in the lymphoid tissues from F1 and F2 mice (Supplementary Figure 4). The analysis of the rearranged *V*_*H*_*DJ*_*H*_ sequences showed that the major parental expanded clone (IgA) found in spleen and ascites of the donor mouse was the only clone detected in the F1 and F2 allotransplanted mice (Table 2). A similar result was obtained when lymphocytes from mouse #20 were used for allo-transplantation. Mouse #20 developed a monoclonal IgM neoplasia consistent with DLBCL. The transfer of splenocytes or ascitic lymphocytes from this mouse into recipient SCID/NOD mice resulted in the expansion of the parental expanded clone (Table 2) and the development of a lymphoma that recapitulated the characteristics of the parental neoplasm (Figures 2F,G). Finally, we also allo-transplanted lymphocytes of mouse #39, which developed lymphadenopathy and ascites, consistent with a plasma cell neoplasia caused by the expansion of an IgA clone (Supplementary Figure 3). Although in this case the tumor implantation took longer, the recipient immunodeficient mouse developed the same neoplasm and clonal IgA expansion than the donor mouse (Table 2).

**Table 2:** TRAF3/BCL2 +/+ B cell neoplasms can be transferred to and survive in immunodeficient mice.

**Mouse ID**	**Source of implanted lymphocytes**	**Days after implant**	**Spleen (mg)**	**Nodes (mg)**	**Other lymphoid anomalies**		**EXPANDED CLONE**						
						**mRNA source**	**Ig subclass**	**VDJ families**	**Homology %**	**SHM**	**Clone %**	**HCDR3**	**Pathology assessment**
TRAF3/BCL2 3	–	–	1200	5000	Ascites	Ascites	IgA	VH3/D2/JH2	93.8	M	62	ASRYGLFDY	HIGH GRADE B-NHL
						Spleen	IgA	VH3/D2/JH2	93.8	M	80	ASRYGLFDY	
						node	IgM	VH12/D2/JH2	97.6	NM	40	AGDSDGYWYFDV	Not determined
3 F1 #1	Ascites frozen (60 × 10^6^)	25	200	2000	Ascites	Ascites	IgA	VH3/D2/JH2	94.4	M	100	ASRYGLFDY	HIGH GRADE B-NHL
						Node	IgA	VH3/D2/JH2	94.4	M	100	ASRYGLFDY	
3 F1 #2	Ascites frozen (60 × 10^6^)	30	100	4500	Ascites	Ascites	IgA	VH3/D2/JH2	94.4	M	100	ASRYGLFDY	
						Node	IgA	VH3/D2/JH2	94.4	M	100	ASRYGLFDY	
3 F2	Ascites 3F1 #1 fresh (60 × 10^6^)	14	200	3000	Ascites	Ascites	IgA	VH3/D2/JH2	94.4	M	100	ASRYGLFDY	
						Node	IgA	VH3/D2/JH2	94.4	M	100	ASRYGLFDY	
TRAF3/BCL2 20	–	–	2470	975	Ascites, pleural effusion	Spleen	IgM	VH5/D4/JH1	96.9	M	100	ARLDWYFDV	DLBCL
						Pleural effusion	IgM	VH5/D4/JH1	96.2	M	100	ARLDWYFDV	
20 F1 #1	Spleen frozen (50 × 10^6^)	153	1800	500	Ascites, pleural effusion	Spleen	IgM	VH5/D4/JH1	96.5	M	100	ARLDWYFDV	
						Node	IgM	VH5/D4/JH1	96.5	M	100	ARLDWYFDV	
						Ascites	Ig	VH5/D4/JH1	96.2	M	100	ARLDWYFDV	
20 F1 #2	Pleural effusion frozen (40 × 10^6^)	160	1500	800	Ascites, pleural effusion	Spleen	IgM	VH5/D4/JH1	96.5	M	100	ARLDWYFDV	
						Pleural effusion	IgM	VH5/D4/JH1	96.5	M	100	ARLDWYFDV	
TRAF3/BCL2 39	–	–	375	800	Ascites	Nodes	IgA	VH3/D2/JH4	96.14	M	100	AKHRYDAMDY	PLASMA CELL NEOPLASM
						Ascites	IgA	VH3/D2/JH4	96.14	M	100	AKHRYDAMDY	
39 F1 #1	Nodes (mesenteric) frozen (60 × 10^6^)	232	220	900	Ascites	Nodes	IgA	VH3/D2/JH4	96.14	M	100	AKHRYDAMDY	
						Ascites	IgA	VH3/D2/JH4	96.14	M	100	AKHRYDAMDY	

*Lymphocytes from representative TRAF3/BCL2 double-tg mice were used to assess whether the expanded B cell clones of these mice could be allotransplanted to immunodeficient SCID/NOD mice. Table indicates the mouse ID, the source of the lymphoid tissues used for implantation and how many days took the recipient mice to develop evidence of disease. Table also show the weight of spleen and lymphoid nodes, and whether ascites and pleural effusion was found. The source of the mRNA sample, the Ig class of the expanded clone is also indicated and highlighted (IgM, green; IgA, red). The IGHV, IGHD and IGHJ families that were reorganized in the clone are indicated, according to IMGT/V-QUEST database. The percentage of sequence homology of the VDJ sequence of each clone compared to that of the germinal sequence (GC) is also indicated (% homology) and whether the clone meets the criteria of having been subjected to somatic hypermutation (SHMs) (non-mutated (NM) ≥ 97.5 identities with the GC sequence; mutated (M) < 97.5% identities with the GC sequence). The percentage of the VDJ sequences analyzed from a given sample corresponding to the expanded clone is indicated (% clone). The HCDR3 of the main clone of mouse #3 (yellow), mouse #39 (pink), and mouse #20 (blue) are highlighted. The B cell neoplasia developed by these representative TRAF3/BCL2 double-tg mice is indicated. The diagnostic was based on macroscopic and microscopic features, flow cytometry and immunohistochemical analysis*.

### *TRAF3*-tg and *TRAF3/BCL2* Double-tg Mice Have a Large Representation of B Cells With Rearranged HCDR3 With Similarities to Those Recognizing PAMPS and DAMPs

We analyzed the HCDR3 sequences obtained from the *TRAF3/BCL2* double-tg mice in an effort to determine the potential antigens recognized by these clones (39), making comparisons with HCDR3 sequences obtained from littermates of the other genotypes (wild-type, *TRAF3*-tg, and *BCL2*-tg). Only HCDR3 sequences with ≥75% identities to antigen-matched HCDR3 sequences were considered for these analyses.

**Figure 5:**
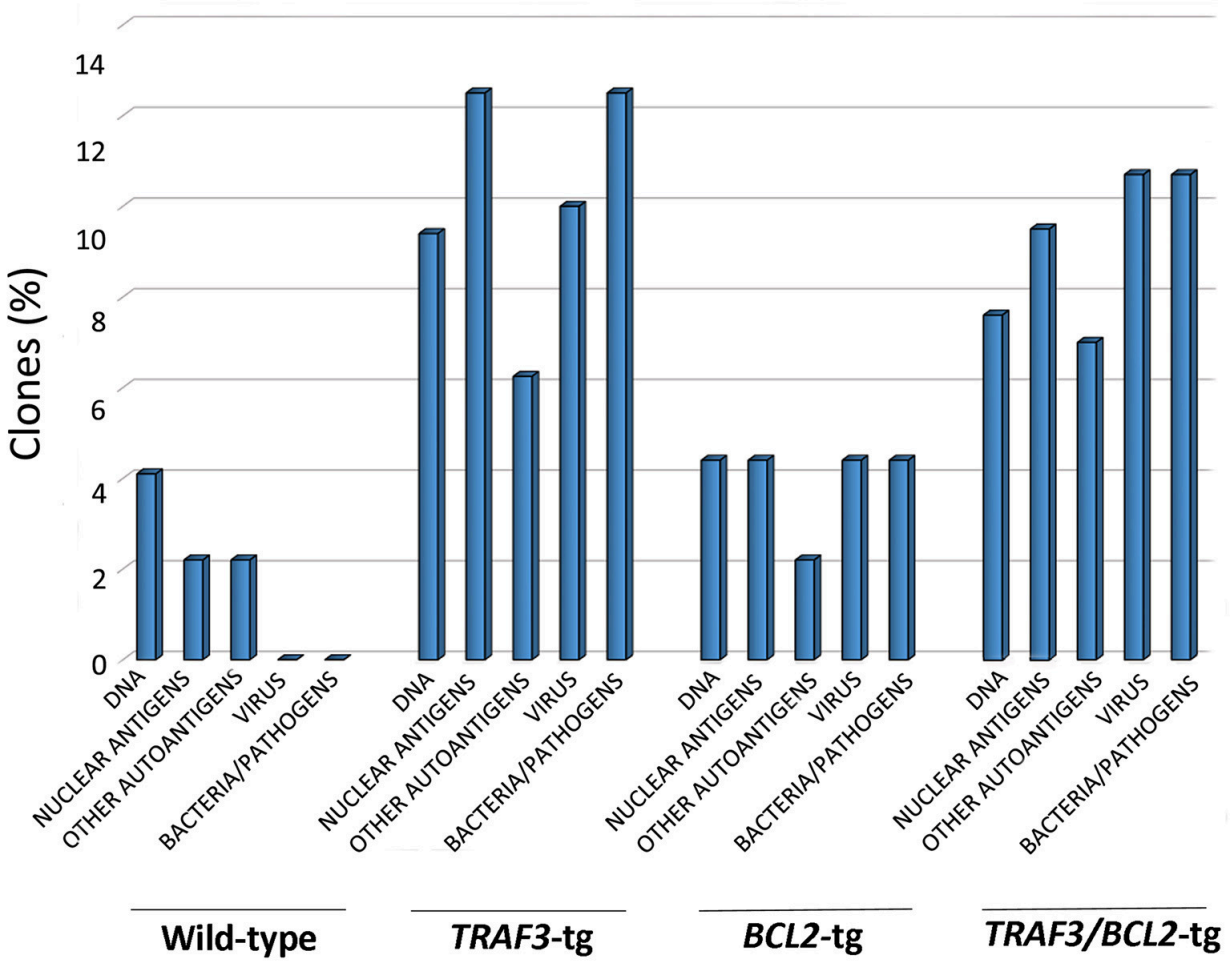
TRAF3 upregulation increases the incidence of immunoglobulins with HCDR3 sequences potentially reactive to autoantigens and PAMPs. HCDR3 sequences were analyzed by blastp (non-redundant protein sequences from *Mus musculus*) and those showing ≥75% identity to described sequences recognizing DNA, ANAs, other autoantigens, and PAMPs (virus, bacteria, and other pathogens) were selected. Data represents the percentage of HCDR3 sequences similar to those recognizing the indicated antigens.

**Figure 6:**
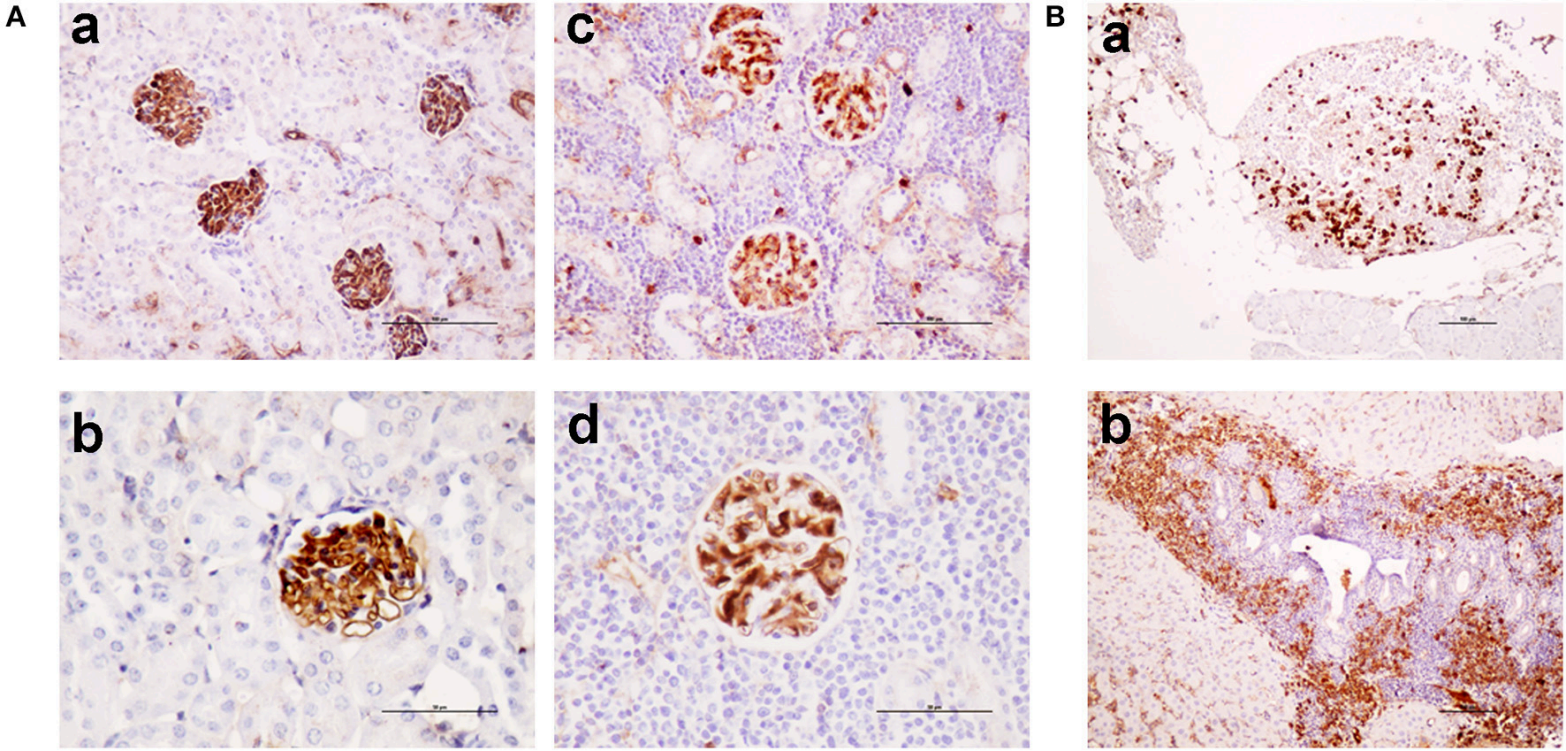
*TRAF3/BCL2* double-tg mice develop autoimmune features similar to the *TRAF3*-tg mice. **(A)** IgG depositions in glomeruli of *TRAF3/BCL2* double-tg mice in samples showing an otherwise normal kidney architecture [**(a)**, 100 x and **(b)**, 200 x] or showing heavy lymphocyte infiltration [**(c)**, 100 x and **(d)**, 200 x]. Microphotographs are from 4 representative *TRAF3/BCL2* double-tg mice. Staining was performed with anti-mouse IgG-HRP. Scale bars are shown [**(a,c)**, 100 μm; **(b,d)**, 50 μm] **(B)**. Tertiary lymphoid organs formation in the *TRAF3/BCL2* double-tg mice. Two representative examples of tertiary lymphoid organs developed in two mice are shown. Panel **(a)** shows a tertiary lymphoid organ in the omentum. Plasma cells are shown by staining with anti-mouse IgG-HRP. Panel **(b)** shows lymphoid neogenesis in the liver, with a prominent presence of plasma cells. Staining was performed with anti-mouse IgG-HRP. Magnification was 100 x. Scale bars are shown (100 μm).

As shown in Figure 5, *TRAF3*-tg and *TRAF3/BCL2*-double-tg mice have a remarkable percentage of *V*_*H*_*DJ*_*H*_ rearrangements producing HCDR3 potentially recognizing DAMPs (including nuclear antigens, DNA), and PAMPs (including phosphatidylcholine, lipoteichoic acid and other bacteria, mite and virus antigens). In contrast, the representation of HCDR3 sequences recognizing these types of antigens is much reduced in wild-type and *BCL2*-tg littermates, thus underscoring the key role of TRAF3 in promoting humoral responses against these antigens. Remarkably, a highly represented group of HCDR3 sequences found in the *TRAF3*-tg and *TRAF3/BCL2* double-tg mice (12.5 and 9.5% of the clones, respectively) had high similarities to HCDR3 recognizing anti-nuclear antigens. The presence of clones expressing autoreactive Ig, such as anti-nuclear antibodies (ANAs) is also consistent with the involvement of TRAF3 in the development of autoimmune disorders and confirms previous results showing the existence of ANAs in the serum of the *TRAF3*-tg mice (14). The expanded *TRAF3/BCL2* double-tg clones maintained this trend and have HCDR3 sequences similar to those recognizing viral antigens, nuclear antigens, DNA and phosphatidylcholine (Figure 5). Consistent with the role of some of these antibodies in autoimmunity, *TRAF3/BCL2* double-tg mice also develop autoimmune lesions, such as IgG depositions in glomeruli and tertiary lymphoid organs formation (Figure 6).

## Discussion

In this report, we show that TRAF3 and BCL2 cooperate to promote development of a variety of mature B cell lymphomas arising from antigen-challenged B cells. In this process, TRAF3 seems to promote antigen-dependent B cell differentiation toward ASCs, and BCL2 seems to provide the survival tools required to facilitate B cell transformation and survival of the expanded clones. Neither TRAF3 nor BCL2 alone have the capacity to support B cell transformation to the same extent that is achieved when both TRAF3 and BCL2 act in combination. Indeed, *BCL2*-tg mice have been shown to develop FL with advanced aging at an approximately 15% incidence (35), although these mice are otherwise healthy and have a normal life-span. In contrast, *TRAF3*-tg mice develop several pathologies associated to inflammation and autoimmunity, including inflammation-driven solid tumors, but very rarely develop lymphoid malignancies (14).

As shown in this report, lymphoid-specific *TRAF3/BCL2* double-tg mice develop B cell neoplasms, mostly DLBCL and plasma cell neoplasia, with high incidence (approximately 80% of the mice). However, the fact that these B cell malignancies arise in mice well over 1 year old suggests that TRAF3 and BCL2 might be necessary but are not sufficient for B cell transformation, and that additional transforming events are required. Nevertheless, given the high incidence of B cell tumors developed by the *TRAF3/BCL2* double-tg mice, the overexpression of both transgenes might favor the occurrence of these additional transforming events. In this regard, upregulation of c-MYC expression has been observed in two *TRAF3/BCL2* double-tg mice that have developed high grade B NHL.

It is noteworthy that many of the B cell lymphomas arising in the *TRAF3/BCL2* double-tg mice show a nuclear localization of TRAF3. Recently, studies showed that TRAF3 can traffic into the nucleus where it associates with and inhibits the transcriptional regulator cAMP response element binding protein (CREB) (35). CREB-binding protein (CREBBP) is a key coactivator of CREB transcriptional function (40) and this gene is frequently mutated in FL and DLBCL (41). Remarkably, mice deficient in *Crebbp* have reduced B cell numbers affecting different B cell subsets. However, BCL2 can overcome these deficiencies and collaborate with *Crebbp* loss to promote DLBCL development, as shown in mice where both *Crebbp* gene inactivation and BCL2 over-expression in B cells were combined (42). Interestingly, c-MYC expression is upregulated and seems to play a crucial role in the B cell transformation process in this mouse model, thus underlining some similarities to the high-grade B NHLs developed by some *TRAF3/BCL2* double-tg mice.

Our results indicate that both lymphocyte-specific *TRAF3*-tg and *TRAF3/BCL2* double-tg mice have a large representation of *V*_*H*_*DJ*_*H*_ rearrangements producing HCDR3 sequences highly similar to those recognizing PAMPs and DAMPs, including DNA, nuclear antigens, and other autoantigens (platelet glycoproteins, hemoglobin and myosin, among others), bacteria antigens (including phosphatidylcholine and lipoteichoic acid), virus, and other parasite antigens. In contrast, wild-type and *BCL2*-tg littermates sharing cages with the *TRAF3*-tg and *TRAF3/BCL2* double-tg and therefore being exposed to the same antigens have significantly fewer of these HCDR3 sequences, thus underscoring TRAF3 involvement in this process. These results are consistent with the participation of TRAF3 in the regulation of several PRRs involved in the innate immune responses to PAMPs and DAMPs. Indeed, a role for TRAF3 in controlling TLR and RLR-mediated interferon (IFN) responses against virus is well-documented (43, 44) and many examples of viral proteins have been identified that subvert TRAF3 antiviral function by targeting it or by out-competing TRAF3 binding to its signaling partners (45, 46). Furthermore, TRAF3 overexpression in B cells induced exacerbated TLR-mediated antibody responses (14). This is consistent with the role of TLRs in humoral responses against bacteria and other pathogens (47) and with the involvement of TRAF3-binding partner MyD88 in promoting robust TLR-mediated B cell humoral responses to virus (48). However, TLR hyper-responsiveness have been also shown in *Traf3*-deficient B cells (18). These seemingly opposite results might underline different TRAF3 requirements to activate the immune response in distinct B cell types. Of note is that, as we previously reported (14), TRAF3 overexpression does not seems to alter the initiation of the humoral response, since the IgM response to TI and TD antigens is similar in *TRAF3*-tg and wild-type mice. Instead, TRAF3 seems to control later stages of B cell differentiation, such as class switching and SHM and/or the duration of antibody responses, as indicated by the elevated IgG serum levels in the *TRAF3*-tg mice and the increased IgG production seen upon antigen challenge. Indeed, in further support of this idea, *TRAF3*-tg, and *TRAF3/BCL2* double-tg B cells with *V*_*H*_*DJ*_*H*_ rearrangements recognizing typical TI antigens, such as DNA and phosphatidylcholine, have gone through class switching and SHM (not shown). Furthermore, most of the DLBCL and plasma cell neoplasms developed by the *TRAF3/BCL-2* double-tg mice are composed by expanded transformed clones that have also undergone class switching and SHM. This is true even for half of the expanded clones expressing IgM, which also show SHM. In contrast, the B cell neoplasms developed by the B cell-specific *Traf3*-deficient mice (28) were consistent with SBL/CLL and MZL, with over 86% of the expanded cloned having non-mutated *V*_*H*_*DJ*_*H*_ regions (applying the 97.5% identity to the germ line criteria that we have used in our analyses).

Altogether, the present evidence allows speculation about whether TRAF3 overexpression might drive TI-antigen activated B cells through an ASC differentiation program that enforces the production of high-affinity, SHM, class-switched antibodies (49). In this scenario, TRAF3 might facilitate antigen-challenged B cells to escape from the B tolerance surveillance mechanisms resulting in the production of autoreactive Ig clones (49). Indeed, it has been shown that high-affinity SHM IgG autoantibodies exacerbate SLE symptoms compared to IgM autoantibodies (50, 51), which is consistent with the presence of IgG depositions in the renal glomeruli of the *TRAF3/BCL2* double-tg mice. Most interestingly, recent results suggest that B cell intrinsic type 1 IFN keeps BCR signaling beyond the threshold required for effective tolerance (52). As a result, type 1 IFN would contribute to the loss of B cell tolerance and the development of autoreactive B cells into the GC and extra-follicular pathways. Thus, considering the key role of TRAF3 in the promotion of efficient type-1 IFN production in response to pathogen challenges, these results may underlie the role of TRAF3 in the development of the SLE (14). Altogether, these results further emphasize the differences between *Traf3*-defficiency and TRAF3 overexpression in B cell pathophysiology and underscore the need of keeping TRAF3 expression tightly regulated to assure normal B cell homeostasis and humoral responses to antigens.

Finally, while ample evidence exists about the role of deleterious *TRAF3* mutations in the development of human B cell neoplasia [which presumably is the result, at least in part, of the activation of NF-κB2-mediated transcriptional programs (21–28)], little is known about whether TRAF3 upregulation also plays a role in human lymphoid tumorigenesis (53). While genomic analysis has not revealed *TRAF3* gene amplification in lymphoid malignancies, epigenetic mechanisms could contribute to elevated TRAF3 expression. Alternatively, a gain of TRAF3 protein function could instead be caused by modifications of either the expression or the activity of any of the abundant proteins involved in TRAF3 regulation (54, 55). Besides, as shown in this article, TRAF3 overexpression in B cells is not sufficient to induced B cell transformation and requires additional partners to facilitate B cell transformation. In summary, the results presented herein are consistent with a scenario in which TRAF3 overexpression or gain-of-function causes the anomalous selection and differentiation of PRR-co-stimulated B cell clones that in combination with BCL2 over-expression predisposes to malignant B cell transformation.

## Author Contributions

JZ and GP-C designed research; GP-C, SL, MV-C, and JZ performed experiments; GP-C, MA, and JZ analyzed data; JR provided essential reagents; JZ, GP-C, MA, and JR interpreted and discussed the data; JZ wrote the paper.

### Conflict of Interest Statement

The authors declare that the research was conducted in the absence of any commercial or financial relationships that could be construed as a potential conflict of interest.
