# Supplementary material for: Dysregulated TRAF3 and BCL2 Expression Promotes Multiple Classes of Mature Non-hodgkin B Cell Lymphoma in Mice
Source: Front Immunol. 2019 Jan 11;9:3114. doi: 10.3389/fimmu.2018.03114 (PMC6338067; doi:10.3389/fimmu.2018.03114)

SUPPLEMENTARY FIGURES

Dysregulated TRAF3 and BCL2 expression promotes multiple classes of mature Non-Hodgkin B cell lymphoma in mice

Gema Perez-Chacon, Magdalena Adrados, Maria T. Vallejo-Cremades, Sophie Lefebvre, John C. Reed, and Juan M. Zapata*

Supplementary Figure 1.

Immunohistochemical analysis of representative examples of DLBCL developed by the *TRAF3/BCL2* double-tg mice. Immunohistochemical analysis of representative examples of DLBCL developed by the *TRAF3/BCL2* double-tg mice are shown. Tissue slides were stained either with H&E or with antibodies specific for human TRAF3 and BCL2 and for mouse CD45B220, BCL6, MUM1, IgG, Ki67, and PCNA as indicated. Scale bars are shown (50 µm).

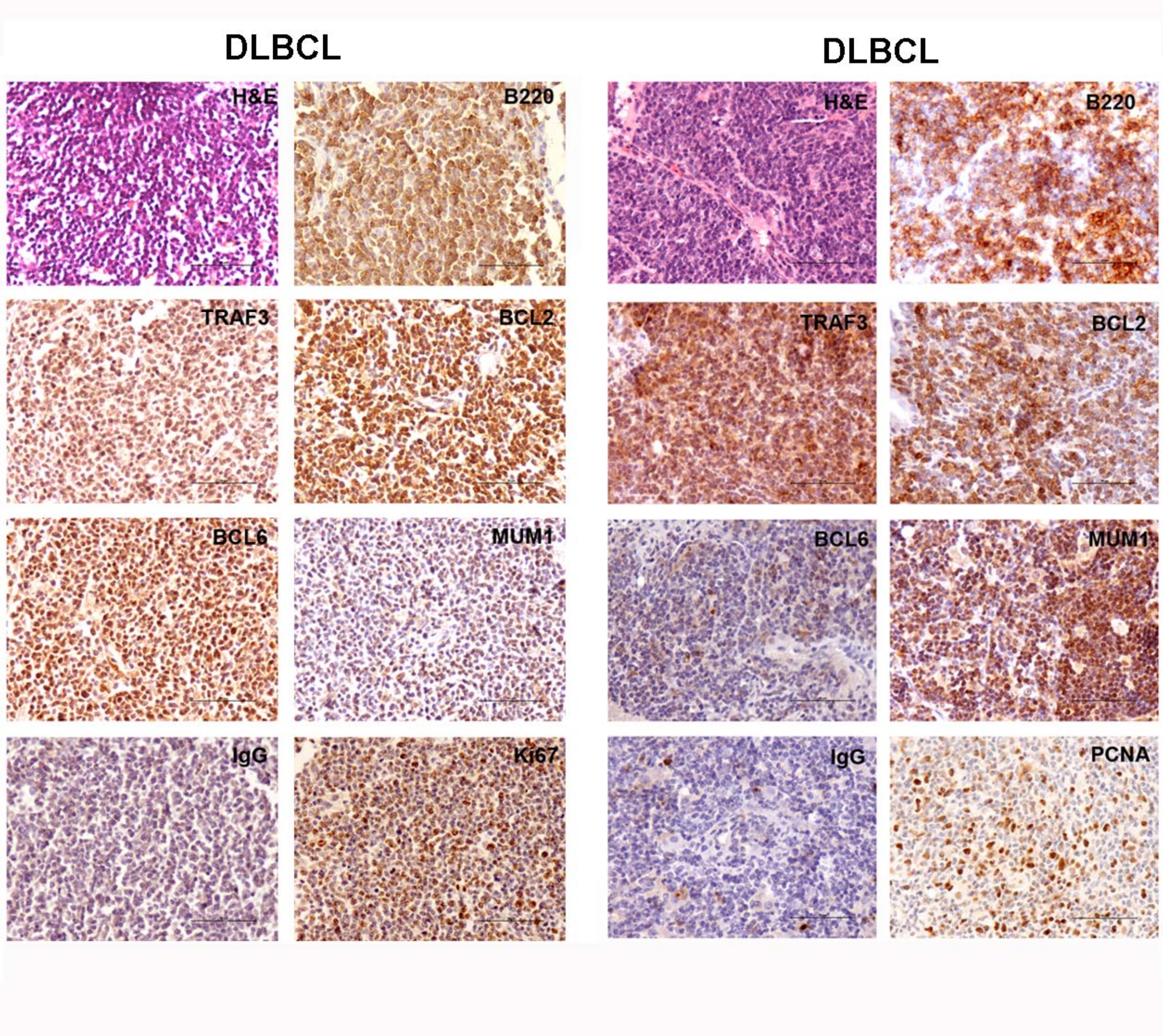

Supplementary Figure 2.

Immunohistochemical analysis of a representative case of High-grade B-NHL developed by *TRAF3/BCL2* double-tg mice. Tissue slides were stained either with H&E or with antibodies specific for human TRAF3 and BCL2 and for mouse CD45B220, BCL6, MUM1, CD10, IgG, and Ki67 as indicated. Magnification was 200x and 600x Scale bars are shown (100 µm).

HIGH GRADE B CEL LYMPHOMA

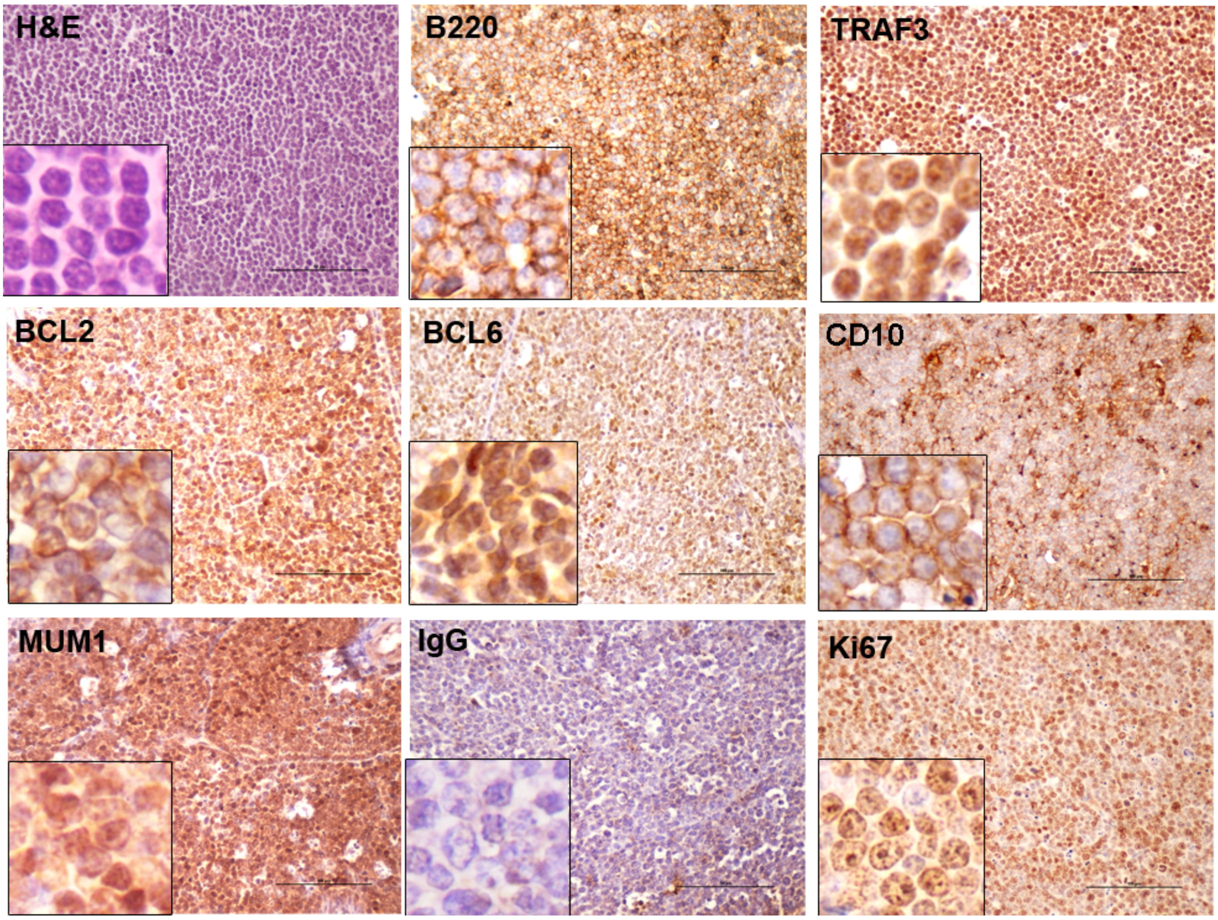

Supplementary Figure 3

Immunohistochemical analysis of representative examples of plasma cell neoplasm neoplasms developed by the *TRAF3/BCL2* double-tg mice. Immunohistochemical analysis of representative examples of IgG (left) and IgA (right) expressing plasma cell neoplasms developed by the *TRAF3/BCL2* double-tg mice are shown. Tissue slides were stained either with H&E or with antibodies specific for human TRAF3 and BCL2 and for mouse CD45B220, BCL6, MUM1, Ki67, and PCNAas indicated. Pre-adsorbed HRP-conjugated anti-mouse IgG or anti-mouse IgA were used to stain the expressed immunoglobulins. Scale bars are shown (50 µm).

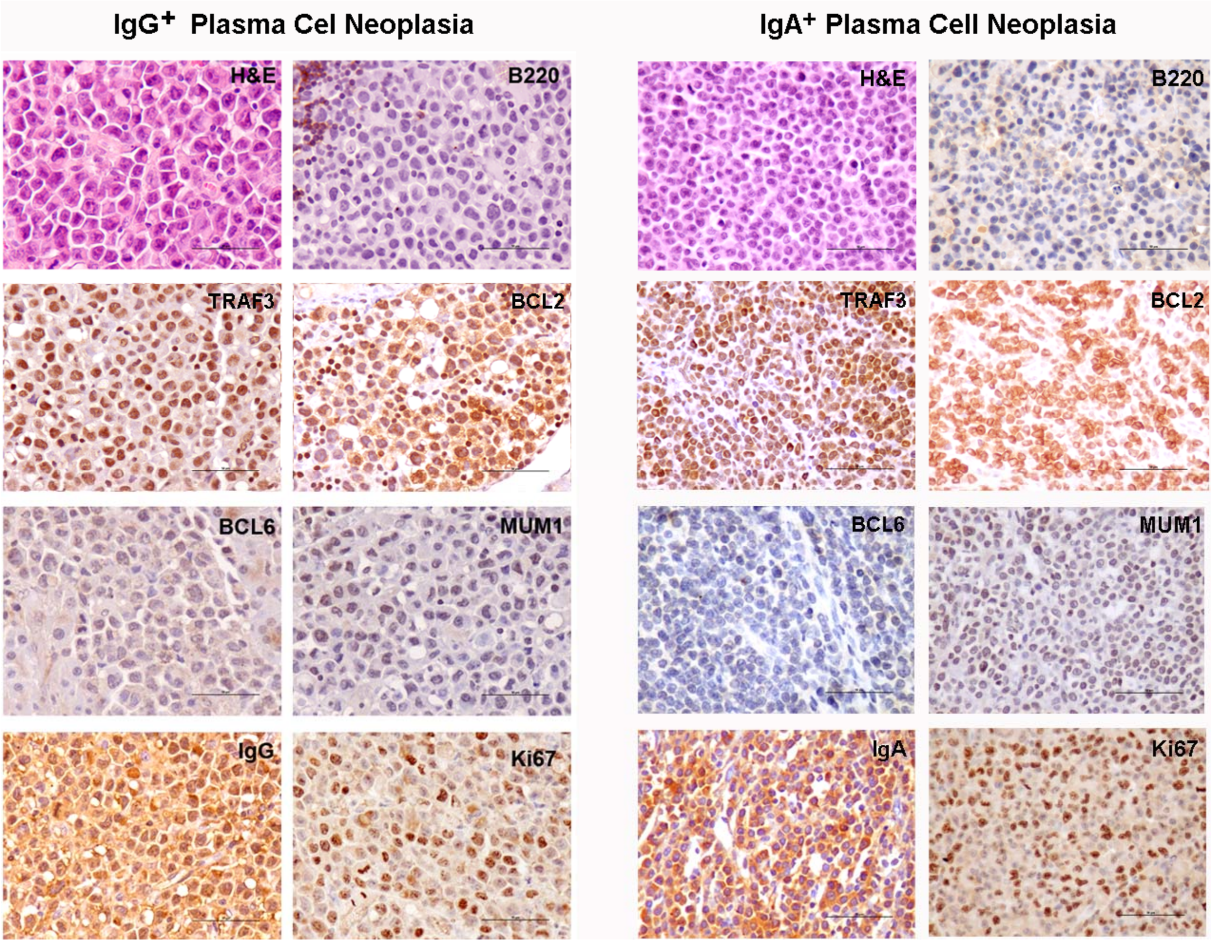

Supplementary Figure 4.

Immunoglobulin subtypes expanded in allotransplanted mice. mRNA from the indicated mice and lymphoid tissues was extracted and retro-transcribed into cDNA using random primers. Then PCR was performed using specific primers for the VDJ region of either IgM, IgG or IgA, as described in Materials and Methods. The amplified PCR fragments were analyzed in 2% agarose gels and staining with Sybr safe and UV light. The position of 1 Kb markers is shown in the first lane.

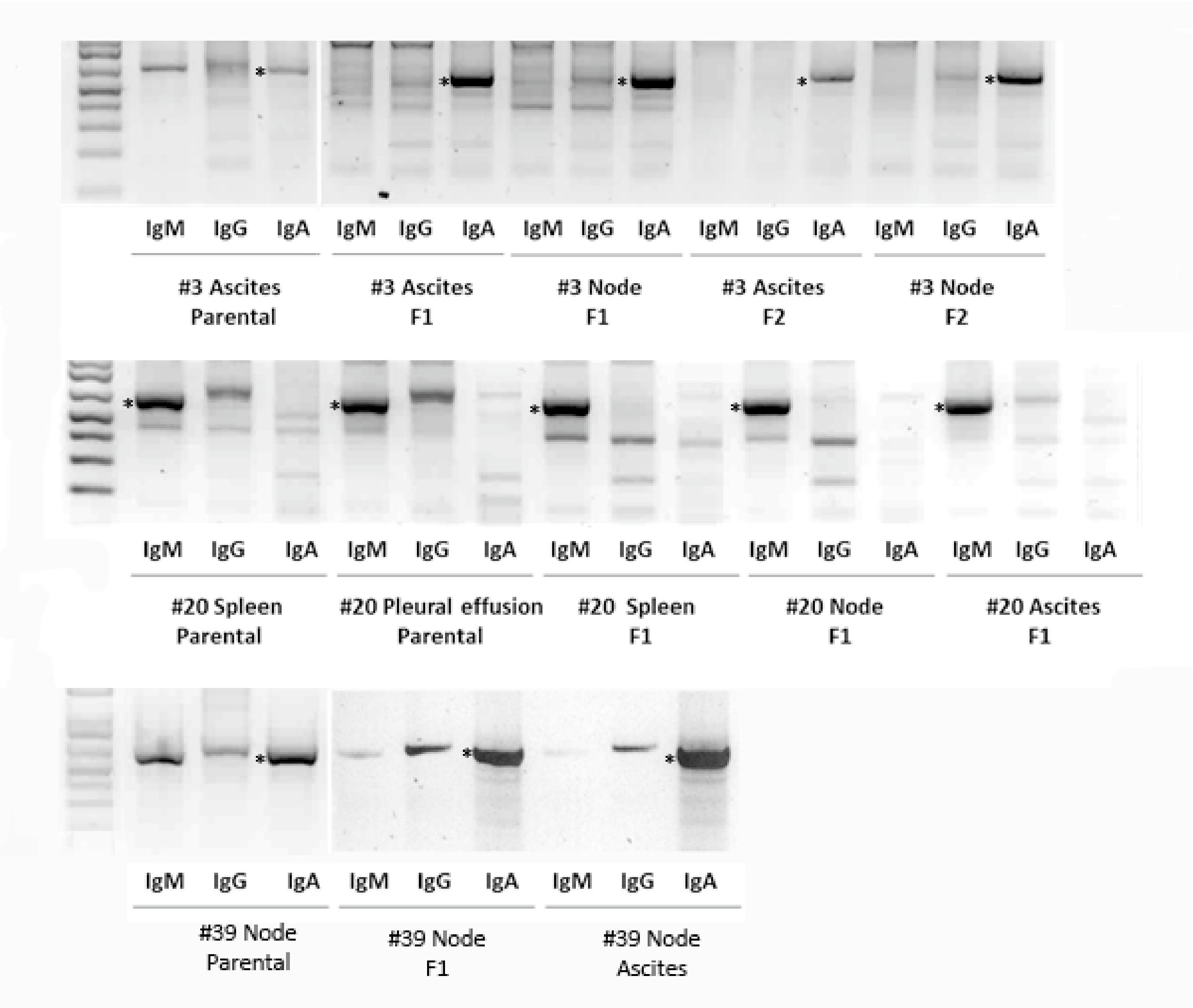

Supplement: Supplementary file 1 [file Data_Sheet_1.PDF]
